# Microbial contamination in water-based metalworking fluid as trigger for occupational hypersensitivity pneumonitis – development of specific IgG tools for a suspected clinical case 

**DOI:** 10.5414/ALX02124E

**Published:** 2020-12-02

**Authors:** Sabine Kespohl, Isabell Warfolomeow, Gerd Schneider, Silke Maryska, Ursula Meurer, Monika Raulf

**Affiliations:** 1 Institute for Prevention and Occupational Medicine of the German Social Accident Insurance, Institute of the Ruhr University Bochum (IPA), Bochum,; 2 German Social Accident Insurance Institution for the Woodworking and Metalworking Industries, BGHM, Mainz, and; 3 Institute for Occupational Safety and Health of the German Social Accident Insurance (IFA), St. Augustin, Germany

**Keywords:** microbial contamination, metal working fluid, hypersensitivity pneumonitis, in-vitro diagnosis, IgG antibodies

## Abstract

Microbially contaminated metal-working fluid (MWF) can cause respiratory symptoms in exposed workers in the form of exogenous allergic alveolitis/hypersensitivity pneumonitis (HP). The diagnosis of HP is based, among others, on the identification of the culprit and the detection of corresponding specific IgG antibodies (sIgG) in the patient’s serum. Commercial antigen tools for the detection of these HP triggers are rarely available; therefore, antigens from contaminated MWF workplace samples were isolated exemplarily for diagnosis of a suspected HP case. Various MWF-specific bacteria were identified in the workplace samples, including *Pseudomonas oleovorans, Pseudomonas alcaliphila, Pseudomonas* spec., *Paenibacillus glucanolyticus,* and *Corynebacterium amycolatum.* The sIgG antigen binding, detected by ImmunoCAP system against MWF antigens from workplace samples and against the identified bacterial antigens, was much stronger in the patient serum compared to selected reference sera. The highest sIgG concentrations in the patient’s serum could be determined against *Pseudomonas* antigens. Inhibition tests showed cross-reactions of MWF and *Pseudomonas* antigens, whereby the *Pseudomonas* antigens cross-reacted less with each other. For in-vitro diagnosis in case of suspected HP caused by contaminated MWF, workplace-related antigens are now available.

**German version published in Allergologie, Vol. 43, No. 1/2020, pp. 26-34**

## Introduction 

According to official mineral oil data for the Federal Republic of Germany, in 2017 ~ 42,000 tons of non-water-based metalworking fluids (MWFs), so-called cutting oils were processed. Additionally, 32,000 tons of water-based MWFs were mixed with a general emulsion ratio of 5 – 8% oil content with water, resulting in a total of 400,000 – 660,000 tons of water-based MWF emulsions [1]. Depending on the process technology (e.g., sawing, grinding, milling, drilling, gear cutting, thread cutting), different processing speeds and lubricating or cooling effects are required. Therefore, MWFs are individually mixed with various additives (e.g., emulsifiers, high-pressure additives, antioxidants, biocides, etc.) and various oils (mineral oil-based oils, oils from synthetic-organic compounds, oils from renewable raw materials) [5]. In addition to type IV sensitization of the skin (MWF contact dermatitis), which can be induced by chemical components such as biocides, emulsifiers, or metal components [10], water-based MWFs can cause allergic-respiratory problems of type III/IV sensitization in the form of hypersensitivity pneumonitis (HP). Mostly symptoms of flu and/or respiratory outbreak were reported in exposed employees 6 – 12 hours after exposure and can mostly be attributed to microbial colonization of MWF. 

The diagnosis of acute/subacute and chronic HP can be established if six diagnostic features are fulfilled. These include detection of an offending antigen exposure, exposure- and/or time-dependent occurrence of symptoms, elevated specific IgG antibodies titer to an appropriate antigen in serum, inspiratory crackles on physical examination, high-resolution computed tomography pattern of HP, and decreased oxygen saturation at rest and/or under exercise or limited diffusion capacity. If all six criteria are fulfilled, an HP is manifest. If one of the above criteria is missing, it may be replaced by one of the following: lymphocytosis in bronchoalveolar lavage (BAL), histopathological findings of the lung compatible with HP, improvement after avoidance of suspected exposure, positive inhalation exposure or provocation test. These diagnostic criteria were defined in 2007 by the HP working group of the German Society for Pneumology and Respiratory Medicine (DGP) and the German Society of Allergology and Clinical Immunology (DGAKI) [26] and were largely adopted in a current position paper of the European Academy of Allergy and Clinical Immunology (EAACI) for occupational HP [19]. 

The detection of causative triggers/antigens as well as specific IgG in serum are therefore important criteria for both diagnosis and assessment of the course of disease. The comprehensive spectrum of known or common HP antigens has been published in detail [18, 25, 27]. The most common sources of HP antigens are animal proteins (globulins/feathered albumin, trigger in avian lung), while metal salts and low-molecular chemicals (isocyanates [24], phthalic anhydrides, pharmaceuticals, and antibiotics) occur in the occupational environment. Rather rarely described are plant proteins (e.g., cabreuva wood [2]) as triggers of HP. In contrast, moulds and bacteria are frequently described as elicitor of HP. With regard to molds especially occupational exposure is known as potential HP triggers, e.g., in garbage-workers [11], during building-restoration, during cheese-sausage production [17], and in agriculture. Bacterial antigens are often co-localized with mold antigens but could be also detected solely, e.g., in humidifier systems, in hot tubes and tabletop fountains [14], and MWFs [16]. In case of MWF-HP, the most frequent antigens arise from bacterial contamination of water-based MWFs. In a study investigating 100 samples of water-based MWFs with additional preserving agents, microbial colonization (> 10^2^ to > 10^7^ cfu/mL) could be measured in 60% of the samples [7]. 

Although some, partly potent, MWF antigens are known, the selection of commercially available IgG antigens is very limited. In this paper, the different steps of antigen identification and testing in the context of in vitro diagnosis are described on the basis of a suspected HP case report of an exposed worker. 

## Production of in-vitro diagnostics from MWF workplace samples 

A 38-year-old male employee complained of typical symptoms of HP after several years of working as a machine operator in a metalworking company. Consequently, information with respect to suspicion of occupational HP was reported. In the course of diagnostic workup, specific IgG antibody concentration against antigens from the MWF samples should be measured in the serum of the exposed patient. 

For this purpose, a total of four MWF samples were taken from different work processes or machines by an occupational safety specialist from the Institute for Occupational Safety of the German Social Accident Insurance (IFA). The microbiological analysis was carried out by cultivation on casein-soya-peptone-agar (CASO) plates at IFA. 

**Figure 1.**
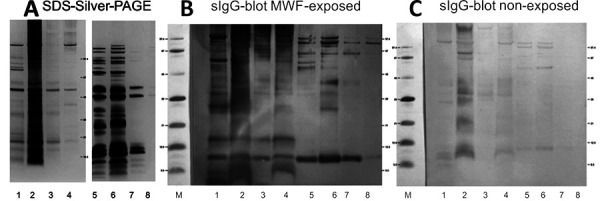
Proteins extracted from four metalworking fluid (MWF) samples. A: Silver stain of SDS-PAGE; B: sIgG blot with serum of MWF-exposed patient; C: sIgG blot with non-exposed reference serum. Lane 1 – 4 proteins from MWF pellet fraction (MWF-13 – MWF-16), lane 6 – 8 proteins from MWF supernatant fraction (MWF-13 – MWF-16).

In parallel, antigen preparation of MWF samples was conducted at the Institute for Prevention and Occupational Medicine of the German Social Accident Insurance (IPA). The MWF samples were sedimented, supernatants were removed and stored for further antigen preparation (supernatant fraction). MWF pellets were suspended with extraction buffer and subsequently treated by ultrasonic bath to disrupt cell membranes of microbial material. Cell debris and insoluble particles were settled by further centrifugation, extracted antigens remained soluble in the buffer (MWF pellet fraction). On the other hand, antigens from four MWF supernatants were precipitated with 80% acetone (final) and re-suspended in phosphate buffer (MWF supernatant fraction). The biochemical analysis of the extracted antigens (pellet and supernatant fraction) included the quantification of the proteins by modified Bradford method and the qualitative analysis of the proteins in SDS-silver-PAGE. All four MWF workplace samples (MWF pellet fraction and MWF supernatant fraction) contained antigens that were visible in silver-stained gel electrophoresis in the range of 5 – 100 kDa (Figure 1A). 

The qualitative antigen characterization was performed as described [12] by sIgG blot with serum of the occupationally exposed patient and a non-exposed subject as reference (Figure 1B, C). Clear IgG binding to antigens was seen with serum of the MWF-exposed patient (Figure 1B), with dominant markers of the protein bands at 10, 15, 27, 60, and 100 kDa and further protein bands in the pellet and supernatant fractions. The reference serum tested for the same protein extracts (Figure 1C) showed only weak IgG bands on proteins of the MWF pellet fraction. Overall, serological IgG binding of the exposed subject was significantly stronger compared to the reference serum. 

The IgG binding strength to antigens from pellet and supernatant fractions was comparable in patient serum, therefore both fractions were combined, biotinylated according to a standard method [23], and coupled to the ImmunoCAP solid phase via biotin-streptavidin affinity. The measured sIgG response in patient serum was stronger on MWF samples 13 and 14 than on MWF samples 15 and 16 (Table 1). The sIgG concentrations against three of the four samples were significantly higher with 77 – 141 mg_A_/L in the patient serum compared to the reference serum with 5.5 – 5.7 mg_A_/L. The highest sIgG response was measured in MWF samples 13 and 14. 

## Identification of MWF-specific bacteria 

The microbial analysis of the four MWF workplace samples was carried out after cultivation at IFA. Clearly identified microorganisms (DNA sequencing or ld Maldi sequencing) were then cultivated and harvested for antigen preparation on at least ten CASO agar plates each. The bacterial material was prepared for antigens in the IPA using Precellys lysing kit (SK38) and ultrasonic bath to prepare soluble proteins as described [12]. The qualitative and quantitative protein and antigen analysis of the bacterial isolates was performed as described above for the MWF antigens. 

**Figure 2.**
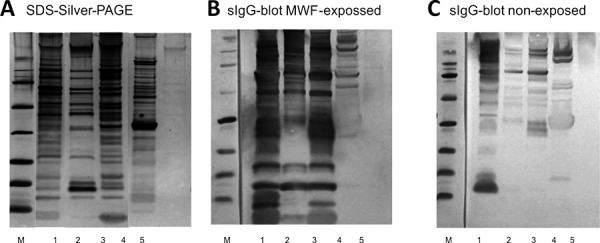
Proteins extracted from metalworking fluid (MWF) bacterial isolates. A: Silver stain of SDS-PAGE; B: sIgG blot with serum of MWF exposed patient; C: sIgG blot with non-exposed reference serum. lane 1: *Pseudomonas oleovorans*, lane 2: *Pseudomonas alcaliphila*, lane 3: *Pseudomonas spec*., lane 4: *Paenibacillus glucanolyticus*, lane 5: *Corynebacterum amycolatum*.

The following prominent bacterial species were identified: *Pseudomonas oleovorans*, *Pseudomonas alcaliphila*, *Pseudomonas* spec., *Paenibacillus glucanolyticus,* and *Corynebacterium amycolatum*. The amount of extractable proteins was in the range of milligrams with protein concentrations between 1 and 2 mg/mL. An exception was *Corynebacterium amycolatum*, here only microgram amounts could be extracted. The protein spectra of the bacteria were individually different in the molecular weight range of 5 – 100 kDa (Figure 2A). A qualitative IgG detection of the bacterial antigens by immunoblot with the serum of the MWF-exposed patient showed a significantly stronger sIgG antigen binding compared to a reference serum (Figure 2 B, C). Overall, the sIgG binding strength was stronger against *Pseudomonas* species than against *Paenibacillus* and *Corynebacterium*. This was seen in both sera independent of exposure. The highest sIgG concentration (Table 2) – determined with the ImmunoCAP system – was measured in the patient serum against *Pseudomonas alcaliphila* with 615 mg_A_/L. In comparison, the maximum value and 95% quantile value of a newly tested reference collective including n = 20 healthy adults were 15.56 and 15.37 mg_A_/L, respectively. 

## Analysis of cross-reactivity among MWF and bacterial antigens 

Potential cross-reactions of antigens and bacterial isolates were investigated by ImmunoCAP inhibition tests. Therefore, one antigen was coupled to ImmunoCAP solid phase, and sIgG binding in patient serum was inhibited by pre-incubation of another antigen in liquid phase (so-called inhibitor). The reduction of sIgG binding to antigen solid phase was calculated compared to pre-incubation with PBS instead of inhibitor. 

**Figure 3.**
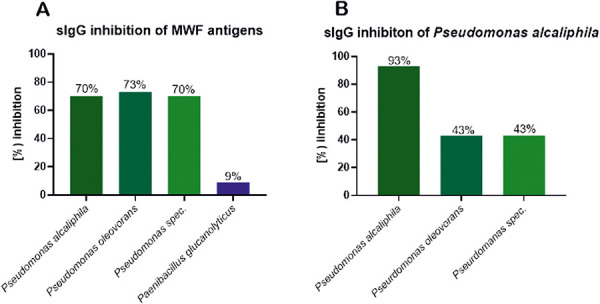
sIgG inhibition test with patient serum. A: Metalworking fluid antigens (solid phase) with different bacterial antigens as inhibitors; B: *Pseudomonas alcaliphila* antigens (solid phase) with different *Pseudomonas* antigens as inhibitor.

Specific IgG binding to MWF antigens (from sample 14) was reduced each by at least 70% by pre-incubation of serum with *Pseudomonas* antigen extracts (Figure 3A). This can be seen as evidence for *Pseudomonas* contamination in MWF sample. However, since the sIgG binding of the patient serum was not completely inhibited by a single *Pseudomonas* species, an additive antigen effect could be possible or additional, not yet identified antigens are included in MWF samples. With *Paenibacillus* antigens as inhibitors, sIgG binding to MWF antigens was reduced by only 9%. This indicates that microbial cultivation alone does not provide any indication of the quantitative composition of the MWF antigens. There could be confounders in selection of culture media conditions or cultivation period that promote growth of some microbial species but is sub-optimal for other species. 

Due to the low protein concentration of the *Corynebacterium* antigen extract and also the low sIgG concentration in the serum of the MWF exposed to *Corynebacterium* antigens, this inhibition approach was not tested. 

The most prominent sIgG antigen of the MWF exposed was *Pseudomonas alcaliphila*. Inhibition with other *Pseudomonas* antigens showed a 43% reduction in sIgG binding for both *Pseudomonas oleovorans* and *Pseudomonas spec*. Thus, cross-reactivity of the Pseudomonas antigens was shown in the MWF-exposed patient, but was below 50%. It could be concluded that the amount of 10 µg inhibitor for autoinhibition of *P. alcaliphila* to *P. alcaliphila* solid phase (15 µg/ImmunoCAP) was sufficient for a complete reduction (> 80%), but the same amount of 10 µg was not sufficient for inhibition with other *Pseudomonas* species. This suggests that different sIgG epitopes are present on different *Pseudomonas* antigens. 

## Discussion 

The preparation of soluble proteins from water-based MWF workplace samples showed that potential IgG antigens were present. The quality of MWF and bacterial antigens was determined by sIgG immunoblot. But why is it important to quantify concentration of sIgG in patient serum? In principle, the presence of sIgG antibodies is not a marker for the disease per se, and absence of sIgG antibodies does not exclude an HP. However, an increased sIgG concentration in patient serum compared to reference serum/reference collective indicates exposure. With corresponding clinical symptoms of an HP and a positive sIgG result, the probability of an HP is higher in an exposed patient compared to a non-exposed patient [9, 15]. 

In order to make a valid statement regarding measured sIgG concentrations, a well characterized reference collective is important [20]. In the suspected HP case presented here, 20 randomized control sera from a sIgG reference value study [20] were tested for MWF bacterial isolates. The sIgG concentration on *Pseudomonas alcaliphila* measured in patient serum was 30 times higher than the maximum value or the 95% quantile value of the reference collective and can therefore be evaluated as a positive criterion for an MWF HP. Based on a reliable diagnosis of HP, tailored therapies and prevention strategies can take place [13]. Quantitative test systems such as ImmunoCAP, Immulite, and Sandwich ELISA are usually more sensitive compared to exclusively qualitative test systems such as Ouchterlony [25, 18]. However, the sIgG concentrations of different test systems should never be compared directly, as there are significant, test-specific differences [30]. 

*Pseudomonas fluorescens* and *Pseudomonas aeroginosa*, *Ochrobactrum anthropi*, *Actinobacter lwoffii*, *Mycobacterium immunogenum*, *Mycobacterium chelonae* and *Mycobacterium gordonae* have been described as triggers of HP in exposure to MWF [15, 18, 25]. *Pseudomonas* and *Mycobacteria* antigens were found to be particularly dominant in MWF antigens [3, 22, 27, 28, 29, 32]. In the present case, no *Mycobacteria* were identified, but pseudomonads (*Pseudomonas oleovorans, Pseudomonas alcaliphila, Pseudomonas spec.*) were identified by microbial cultivation and subsequent mass spectrometric analysis. High concentration of sIgG in patient serum against *Pseudomonas* antigens as well as strong inhibition with *Pseudomonas* antigens on MWF demonstrated the dominant role of *Pseudomonas* antigens. 

How often do *Pseudomonas species* occur in workplace samples of water-based MWF? The investigation by Dilger et al. [7] showed that ~ 70% of identified microbial contaminations in MWF samples were *Pseudomonas* species. A further extensive evaluation (summarized in DGUV Information 209-051 [6]) of 1,500 air or MWF samples from exposure database MEGA of IFA also identified pseudomonads as typical MWF germs. *Pseudomonas oleovorans* and *Pseudomonas alcaligenes* were identified in more than 20 MWF samples and *Pseudomonas aeruginosa* in 10 – 19 MWF samples. Mycobacteria of the species *immunogenum, chelonae, gordonae* were identified less frequently (< 10/1,500 samples) in MWF samples. 

The identification of *Mycobacteria* in MWF samples is difficult by classical agar cultivation, but can be optimized with PCR-based test systems [31]. In MWF samples from ten metal processing plants (USA and Canada) examined using PCR-based tests, 95% of samples showed contamination with mycobacteria. However, the detection of exposure to mycobacteria, pseudomonads and other microorganisms alone is not sufficient for a diagnosis of HP. Important is a standardized, cultivation-independent test of relevant MWF HP antigens, and even more precise would be a biochemical identification of individual IgG-binding proteins [21]. In a corresponding study, Roussel et al. [21] identified six IgG-binding proteins from *Mycobacterium immunogenum* and expressed these antigens recombinantly for serological IgG diagnostics. 

However, since normally neither major antigens (mostly proteins or glycoproteins relevant for IgG binding) nor commercial test solutions are known for new or rare antigen sources, it is important to use validated and standardized materials and extraction processes when producing allergen extracts [4, 8]. This starts with optimal cultivation conditions of the bacteria to be tested regarding culture media, cultivation temperatures and times. The extraction should be performed under physiological conditions as far as possible, whereby antigenic proteins must be protected from enzymatic degradation by non-proteinogenic protease inhibitors. In addition, a rapid extraction procedure with appropriate cooling of the extracts should be used. The antigen-protein content should be determined and antigen quality should be examined in SDS-PAGE and by immunoblot. Antigens prepared in this way can then be used in sensitive and quantitative test systems for quantitative determination of sIgG concentrations in patient sera. 

For evaluation of measured sIgG concentrations, there is no general classification, as it is common for IgE concentrations in e.g. CAP-classes, but for each antigen a corresponding reference range has to be determined, as described in [20]. It is important that reference sera are obtained from healthy individuals without exposure to investigated antigens. Furthermore, reference sera should be matched with patient serum regarding sIgG to non-antigenic proteins such as human serum albumin (HSA) or maltose-binding protein (MBP). 

MWF and bacterial antigen extracts and tests prepared for our current HP patient correspond to the above mentioned criteria and are now available for further tests of MWF-induced HP. Additionally, a MWF antigen screening tool was developed and testing can be requested as well via https://www.ipa-dguv.de/ipa/research/baproj-e/index.jsp. 

## Funding 

This study was supported by the DGUV (German Social Accident Insurance, IPA project 145-Bioaerosole, St. Augustin, Germany). 

## Conflict of interest 

All authors declare that there is no conflict of interest regarding this work. 

**Table 1.** Serological results – sIgG concentrations to KSS antigens.

Serum	KSS-13 bg449 IgG (mg_A_/L)	KSS-14 bg450 IgG (mg_A_/L)	KSS-15 bg451 IgG (mg_A_/L)	KSS-16 bg452 IgG (mg_A_/L)	HSA Ro401 IgG (mg_A_/L)
KSS-exposed person	141.00	104.00	76.90	24.00	11.00
Reference serum	5.45	5.61	5.73	5.21	6.16

**Table 2.** Serological results – sIgG concentrations to bacterial isolate antigens.

	*Pseudomonas alcaliphila* bg457 IgG (mg_A_/L)	*Pseudomonas oleovorans* bg471 IgG (mg_A_/L)	*Pseudomonas spec.* bg455 IgG (mg_A_/L)	*Paenibacillus glucanolyticus* bg454 IgG (mg_A_/L)	*Corynebacterium amycolatum* bg456 IgG (mg_A_/L)	HSA Ro401 IgG (mg_A_/L)
KSS-exposed person	615.00	142.00	153.00	29.50	15.40	11.00
Reference collective (n = 20) 95^th^ percentile*	15.37	20.16	12.24	13.03	8.58*	2.75

*Reference serum (n = 1)

## References

[1] Amtliche Mineralöldaten MWV..

[2] Baur X Gahnz G Chen Z Extrinsic allergic alveolitis caused by cabreuva wood dust. J Allergy Clin Immunol. 2000; 106: 780–781. 10.1067/mai.2000.109616 11031353

[3] Burton CM Crook B Scaife H Evans GS Barber CM Systematic review of respiratory outbreaks associated with exposure to water-based metalworking fluids. Ann Occup Hyg. 2012; 56: 374–388. 22267130 10.1093/annhyg/mer121

[4] Chapman MD Ferreira F Villalba M Cromwell O Bryan D Becker W-M Fernández-Rivas M Durham S Vieths S van Ree R Consortium C The European Union CREATE project: a model for international standardization of allergy diagnostics and vaccines. J Allergy Clin Immunol. 2008; 122: 882–889.e2. 18762328 10.1016/j.jaci.2008.07.030

[5] DGUV. ifa/praxishilfen/kühlschmierstoffe..

[6] DGUV. DGUV Information 209-051. Keimbelastung wassergemischter Kühlschmierstoffe. www.dguv.de/publikationen; 2016.

[7] Dilger S Fluri A Sonntag H-G Bacterial contamination of preserved and non-preserved metal working fluids. Int J Hyg Environ Health. 2005; 208: 467–476. 16325556 10.1016/j.ijheh.2005.09.001

[8] Esch RE Manufacturing and standardizing fungal allergen products. J Allergy Clin Immunol. 2004; 113: 210–215. 14767431 10.1016/j.jaci.2003.11.024

[9] Fenoglio C-M Reboux G Sudre B Mercier M Roussel S Cordier J-F Piarroux R Dalphin J-C Diagnostic value of serum precipitins to mould antigens in active hypersensitivity pneumonitis. Eur Respir J. 2007; 29: 706–712. 17182654 10.1183/09031936.00001006

[10] Geier J Kühlschmierstoffe in der Metallverarbeitung. Allergo J. 2019; 28: 16–18.

[11] Hagemeyer O Bünger J van Kampen V Raulf-Heimsoth M Drath C Merget R Brüning T Broding HC Occupational allergic respiratory diseases in garbage workers: relevance of molds and actinomycetes. Adv Exp Med Biol. 2013; 788: 313–320. 23835992 10.1007/978-94-007-6627-3_42

[12] Kespohl S Maryska S Zahradnik E Sander I Brüning T Raulf-Heimsoth M Biochemical and immunological analysis of mould skin prick test solution: current status of standardization. Clin Exp Allergy. 2013; 43: 1286–1296. 24152161 10.1111/cea.12186

[13] Koschel D Neues zur EAA in der Literatur. Allergologie. 2008; 31: 457–459.

[14] Koschel D Stark W Karmann F Sennekamp J Müller-Wening D Extrinsic allergic alveolitis caused by misting fountains. Respir Med. 2005; 99: 943–947. 15950134 10.1016/j.rmed.2005.01.004

[15] Lopata AL Schinkel M Potter PC Jeebhay MF Hashemi C Johansson SGO van Hage-Hamsten M Qualitative and quantitative evaluation of bird-specific IgG antibodies. Int Arch Allergy Immunol. 2004; 134: 173–178. 15153798 10.1159/000078651

[16] Merget R Sander I van Kampen V Raulf-Heimsoth M Rabente T Kolk A Brüning T Hypersensitivity pneumonitis due to metalworking fluids: how to find the antigens. Adv Exp Med Biol. 2013; 788: 335–340. 23835995 10.1007/978-94-007-6627-3_45

[17] Morell F Cruz M-J Gómez FP Rodriguez-Jerez F Xaubet A Muñoz X Chacinero’s lung – hypersensitivity pneumonitis due to dry sausage dust. Scand J Work Environ Health. 2011; 37: 349–356. 21327319 10.5271/sjweh.3151

[18] Nogueira R Melo N Novais E Bastos H Martins N Delgado L Morais A Mota PC Hypersensitivity pneumonitis: Antigen diversity and disease implications. Pulmonology. 2019; 25: 97–108. 30126802 10.1016/j.pulmoe.2018.07.003

[19] Quirce S Vandenplas O Campo P Cruz MJ de Blay F Koschel D Moscato G Pala G Raulf M Sastre J Siracusa A Tarlo SM Walusiak-Skorupa J Cormier Y Occupational hypersensitivity pneumonitis: an EAACI position paper. Allergy. 2016; 71: 765–779. 26913451 10.1111/all.12866

[20] Raulf M Joest M Sander I Hoffmeyer F Nowak D Ochmann U Preisser A Schreiber J Sennekamp J Koschel D Update of reference values for IgG antibodies against typical antigens of hypersensitivity pneumonitis. Allergo J Int. 2019; 28: 192–203.

[21] Roussel S Rognon B Barrera C Reboux G Salamin K Grenouillet F Thaon I Dalphin J-C Tillie-Leblond I Quadroni M Monod M Millon L Immuno-reactive proteins from Mycobacterium immunogenum useful for serodiagnosis of metalworking fluid hypersensitivity pneumonitis. Int J Med Microbiol. 2011; 301: 150–156. 20850379 10.1016/j.ijmm.2010.07.002

[22] Saha R Donofrio RS The microbiology of metalworking fluids. Appl Microbiol Biotechnol. 2012; 94: 1119–1130. 22543351 10.1007/s00253-012-4055-7

[23] Sander I Kespohl S Merget R Goldscheid N Degens PO Bruning T Raulf-Heimsoth M A new method to bind allergens for the measurement of specific IgE antibodies. Int Arch Allergy Immunol. 2005; 136: 39–44. 15591812 10.1159/000082583

[24] Schreiber J Knolle J Sennekamp J Schulz KT Hahn JU Hering KG Raulf-Heimsoth M Merget R Sub-acute occupational hypersensitivity pneumonitis due to low-level exposure to diisocyanates in a secretary. Eur Respir J. 2008; 32: 807–811. 18757705 10.1183/09031936.00060507

[25] Sennekamp H-J Extrinsic allergic alveolitis. Hypersensitivity pneumonitis. Munich – Orlando: Dustri; 2004.

[26] Sennekamp J Müller-Wening D Amthor M Baur X Bergmann K-C Costabel U Kirsten D Koschel D Kroidl R Liebetrau G Nowak D Schreiber J Vogelmeier C [Guidelines for diagnosing extrinsic allergic alveolitis (hypersensitivity pneumonitis) (German Extrinsic Allergic Alveolitis Study Group)]. Pneumologie. 2007; 61: 52–56. 17253211 10.1055/s-2006-944326

[27] Sennekamp J Lehmann E Joest M Berufsbedingte exogen-allergische Alveolitis. ASU Arbeitsmed Sozialmed Umweltmed.. 2015; 50: 38–52.

[28] Thorne PS Adamcakova-Dodd A Kelly KM O’neill ME Duchaine C Metalworking fluid with mycobacteria and endotoxin induces hypersensitivity pneumonitis in mice. Am J Respir Crit Care Med. 2006; 173: 759–768. 16387809 10.1164/rccm.200405-627OC PMC2662953

[29] Tillie-Leblond I Grenouillet F Reboux G Roussel S Chouraki B Lorthois C Dalphin J-C Wallaert B Millon L Hypersensitivity pneumonitis and metalworking fluids contaminated by mycobacteria. Eur Respir J. 2011; 37: 640–647. 20693254 10.1183/09031936.00195009

[30] van Toorenenbergen AW Between-laboratory quality control of automated analysis of IgG antibodies against Aspergillus fumigatus. Diagn Microbiol Infect Dis. 2012; 74: 278–281. 22925654 10.1016/j.diagmicrobio.2012.07.002

[31] Veillette M Pagé G Thorne PS Duchaine C Real-time PCR quantification of Mycobacterium immunogenum in used metalworking fluids. J Occup Environ Hyg. 2008; 5: 755–760. 18821262 10.1080/15459620802446343

[32] Wallace RJ Zhang Y Wilson RW Mann L Rossmoore H Presence of a single genotype of the newly described species Mycobacterium immunogenum in industrial metalworking fluids associated with hypersensitivity pneumonitis. Appl Environ Microbiol. 2002; 68: 5580–5584. 12406752 10.1128/AEM.68.11.5580-5584.2002 PMC129929

